# Rhinocerebral mucormycosis with dissemination to pontine area in a diabetic patient: Treatment and management

**DOI:** 10.1002/ccr3.2255

**Published:** 2019-06-05

**Authors:** Bruno Galletti, Francesco Gazia, Cosimo Galletti, Fulvio Perani, Francesco Ciodaro, Francesco Freni, Francesco Galletti

**Affiliations:** ^1^ Unit of Otorhinolaryngology, Department of Adult and Development Age Human Pathology “Gaetano Barresi” University of Messina Messina Italy

**Keywords:** amphotericin B, endoscopic sinus surgery, mucor, rhinocerebral mucormycosis, zygomycetes

## Abstract

Rhinocerebral mucormycosis is a rapidly life‐threatening disease caused by a fungal infection. Every diabetic patient with sinusitis symptoms, headache, and visual changes needs radiological approach and nasal endoscopy to rule out mucormycosis. The mortality rate is 50%‐85%, despite an early diagnosis and a correct treatment.

## 1 INTRODUCTION

We present a case of a patient of 59‐year‐old with an untreated diabetes who developed Rhinocerebral mucormycosis. He was treated with endoscopic sinus surgery (ESS) and medical treatment with amphotericin B intravenous, intrathecal, and endosinusal administration. Despite an early diagnosis, the patient died 2 weeks after admission.

Mucormycosis is an aggressive, angioinvasive, and often rapidly life‐threatening disease caused by fungal infection. There are seven genera of Mucorales fungi found in human infections: Rhizopus, Mucor, and Rhizomucor, Cunninghamella, Absidia (now Lichtemia), Saksenaea, and Apophysomyces. The Mucorales are ubiquitous, but their exact ecology is unknown. They are found in rotting biological material and are thermotolerant. The disease has a global distribution, and Mucorales infection is influenced by seasonal variation.^1^ This disease, in developed countries, is rare and mostly affects diabetic or immunosuppressed patients or subjects undergoing chemotherapy. Conversely, in developing countries, especially in the Asian subcontinent, mucormycosis affects patients suffering from massive trauma or uncontrolled diabetes.^2^ Mucormycosis remains a rare disease, it is most common in high‐risk patients, and it represents 8.3%‐13% of all fungal infections detected in autopsies.^3^ A recent study in the general population in Spain found an incidence of 0.43/1 000 000 inhabitants and 0.62/100 000 hospital admissions, while the analysis of hospital records in France showed an increasing incidence from 0.7/1 000 000 in 1997 to 1.2/1 000 000 in 2006.^4^, ^5^, ^6^ Rhinocerebral mucormycosis (ROCM) is the most frequent kind of mucormycosis in diabetic patients. The disease starts after inhalation of fungal spores into the paranasal sinuses. The fungal infection quickly spreads into adjacent tissues. At the time of germination, the infection spreads to the cavernous sinus, the palate, and the sphenoid sinus progressively involving the brain and the orbits. The fungus infects the central nervous system through the orbital apex or the cribriform plate of the ethmoid bone and eventually leads to the death of the patient. Hematogenous dissemination of the infection can be promoted by cerebral vascular invasion of the fungus, sometimes with the formation of fungal aneurysms. Ultimately, imaging studies are usually unsatisfactory for ROCM diagnosis, and a correct diagnosis of ROCM is usually carried out by histopathological examination of the hyphae of the Mucorales in samples.^7^ We present a case of ROCM in a diabetic patient, treated with endoscopic sinus surgery (ESS) and medical treatment with amphotericin B intravenous, intrathecal, and endosinusal administration.

## 2 CASE REPORT

**Figure 1:**
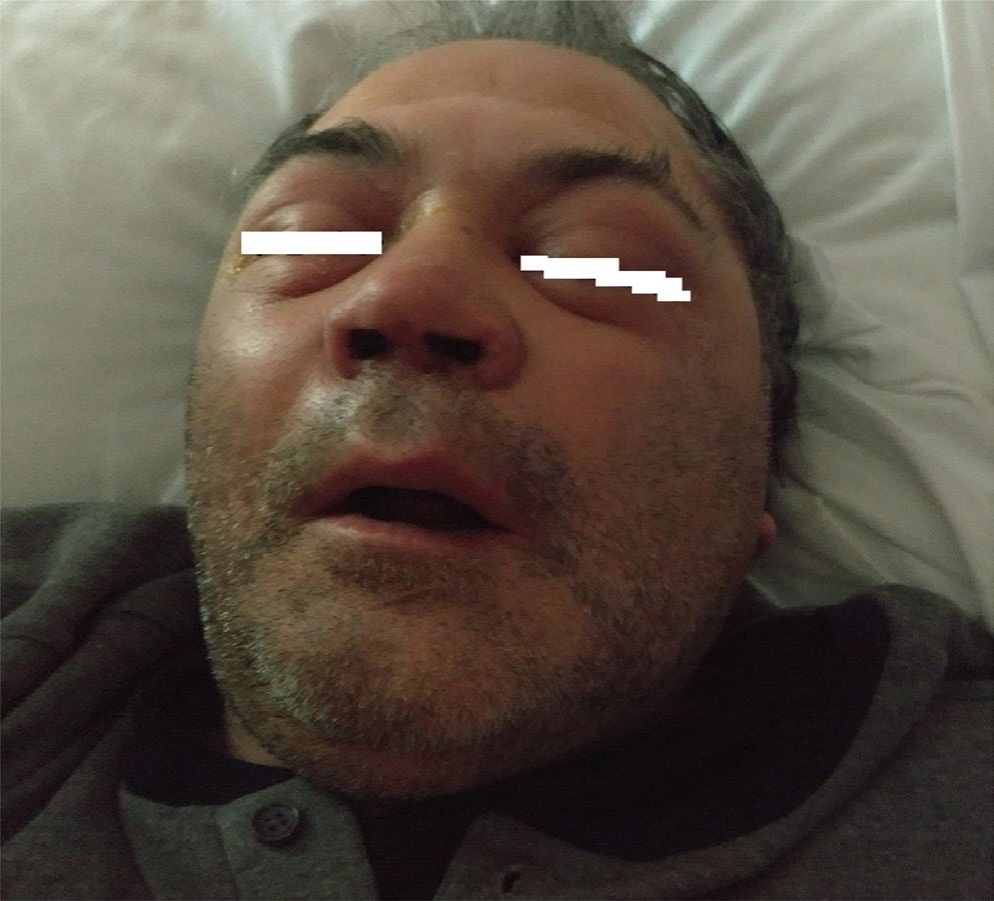
The patient comes to our observation with bilateral periorbital cellulitis

**Figure 2:**
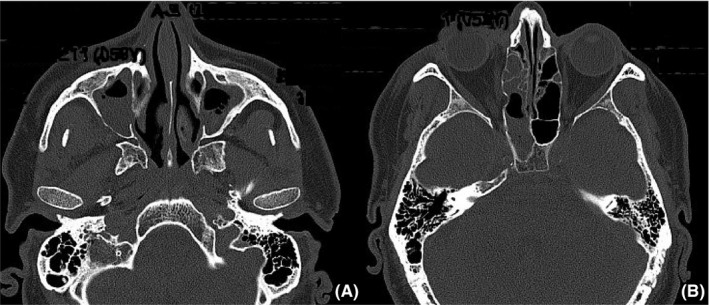
Assial CT study documented opacification of the maxillary sinuses (A), of the right sphenoid sinus and the ethmoidal cells (B)

We present a case of a patient of 59‐year‐old with a family history of diabetes, hypothyroidism,^8^, ^9^, ^10^ and adeno‐tonsillar surgery.^11^ He suffered from benign positional vertigo^12^ and vocal cord cysts.^13^ The patient came to General First Aid with symptoms attributable to sinusitis with bilateral periorbital cellulitis and headache (Figure 1). CT skull study documented widespread opacification and thickening of the maxillary, ethmoidal, and sphenoid sinuses. Moreover, there is a considerable presence of soft tissue swelling in the right periorbital site (Figure 2). The patient is admitted to our otolaryngologist‐operating unit. At the neurological counseling, osteotendinous reflexes appear norm vivacious and symmetrical, without any deficit of the extrinsic ocular muscles and absent meningeal signs. Diabetologic consultation is also performed, since the patient has a sugar level in the blood of more than 600 mg/dL and immediately starts insulin and moisturizing therapy. The patient had misdiagnosed diabetes, so he did not perform any hypoglycemic therapy before hospitalization. The remaining biotemporal examinations are concerned; a leukocytosis with GB over 20 000 is reported. A normal blood gas analysis was obtained.

**Figure 3:**
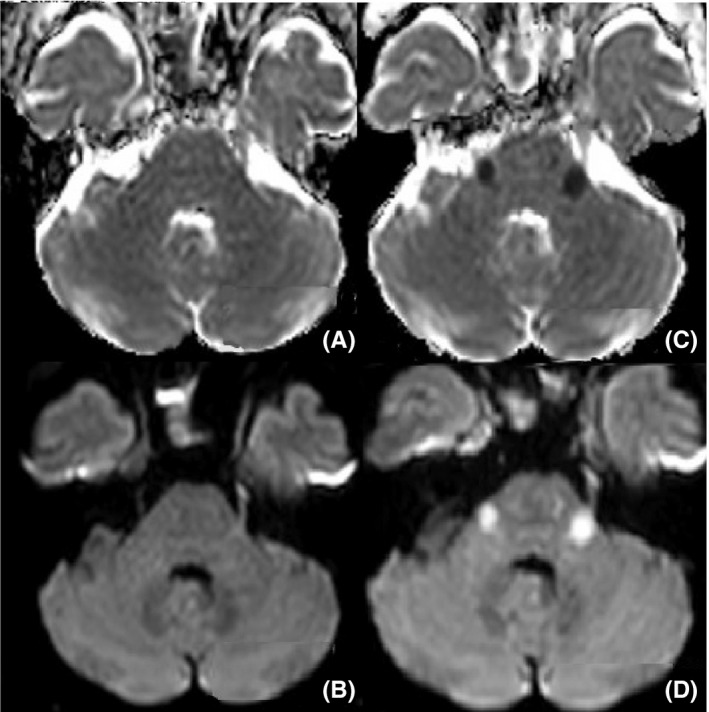
MRI sequences ADC and DWI done before (A; B) and after (C; D) 24 h that documented the appearance of two lesions in bilateral pontine sites, near the trigeminal nerve emergence

**Figure 4:**
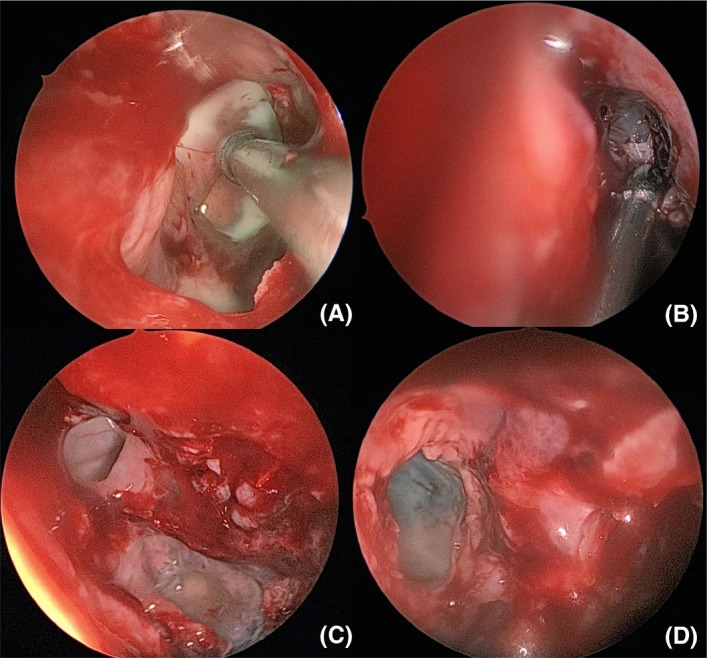
Presence of whitish spongy secretions in right (A) and left (B) maxillary sinuses, in right posterior ethmoid (C) and left sphenoid sinuses (D) during endoscopic surgery

The next day a worsening of the neurological situation has been recorded, with altered facial sensitivity and deficiency of masticatory muscles. Brain MRI is performed with contrast agent that documenting the presence of the known pathological material/tissue in the context of the sinuses. Moreover, diffuse swelling of the lower palpebral soft tissues is appreciated, bilaterally. The muscles of the right infratemporal fossa and the omolateral pterygoid muscles are also partially swollen. Normal condition of the Meckel cave and the cavernous sinuses (Figure 3A,B). Comparing the worsening of the neurological picture, the lack of response to antibiotic therapy and persistent high level of glucose in the blood, a possible fungal infection is suspected. Bilateral ESS was performed without assisted navigation,^14^, ^15^, ^16^ practicing uncibullectomy with a large bilateral meatotomy, bilateral ethmoidectomy, left intranasal sphenoidectomy, and transethmoidal right sphenoidectomy (Figure 4). Pathological tissue has been sent to extemporaneous histopathological examination to search fungal hyphae. The result was negative.

The day after the surgical treatment, there were new worsening of clinical conditions, especially from the neurological point of view, with a paralysis of the III, IV, V, and IV bilateral cranial nerves.

A new MRI documents the appearance of two lesions in bilateral pontine sites, near the trigeminal nerve emergence (Figure 3C,D), pathological tissue in the context of both the Meckel Cave and both cavernous sinuses, increased swelling of the periorbital soft tissues bilaterally.

**Figure 5:**
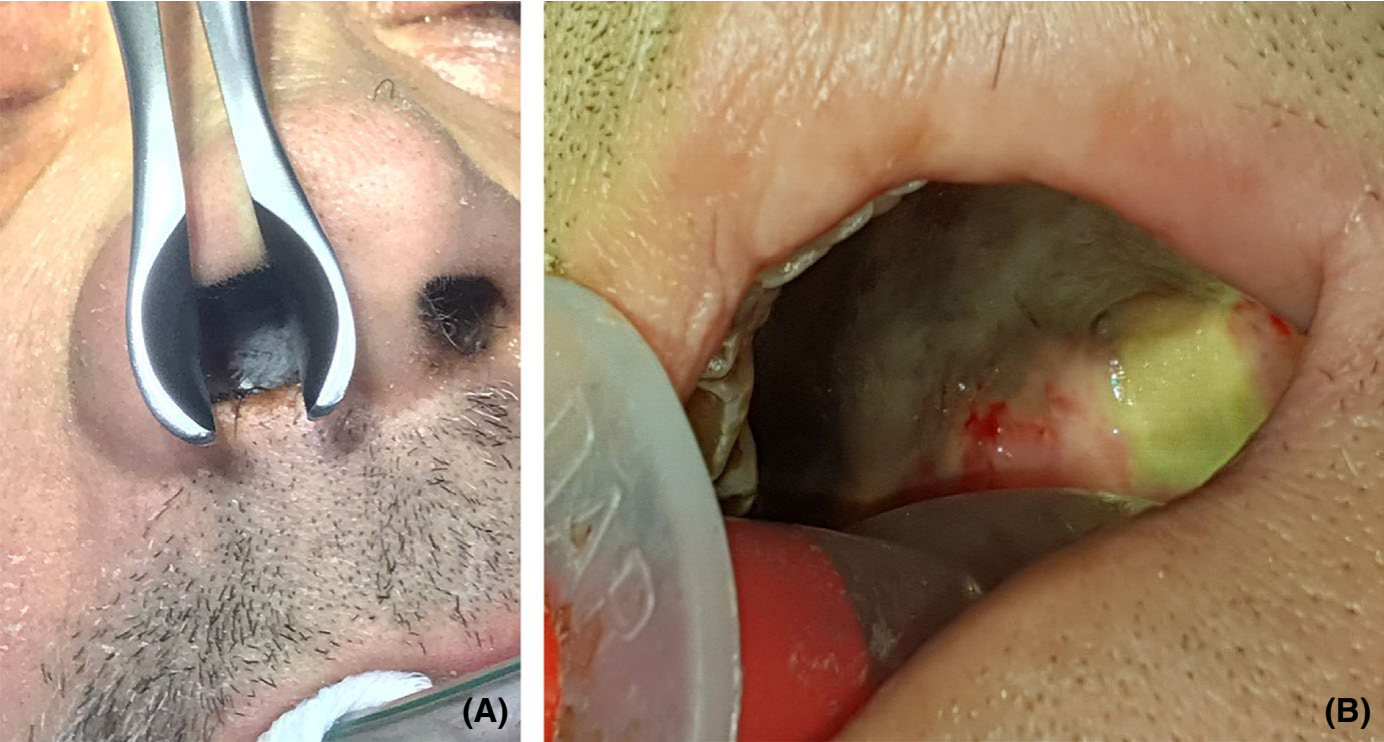
Whitish spongy secretions are present in the nasal cavities (A) and in the oral cavity (B)

Whitish spongy secretions are present in the nasal cavities and in the oral cavity (Figure 5). A new biopsy sampling is performed in the paranasal sinuses, given the strong suspicion of a fungal lesion. This time the histopathological examination is positive for the presence of Mucor's fungal hyphae, order of Mucorales.

The patient is transferred in the intensive care unit and started therapy with Amphotericin B (from 5 to 10 mg/kg/d) intravenously and with paranasal sinuses wash. The patient during the recovery was intubated and stabilized from the hemodynamic and respiratory point of view.

**Figure 6:**
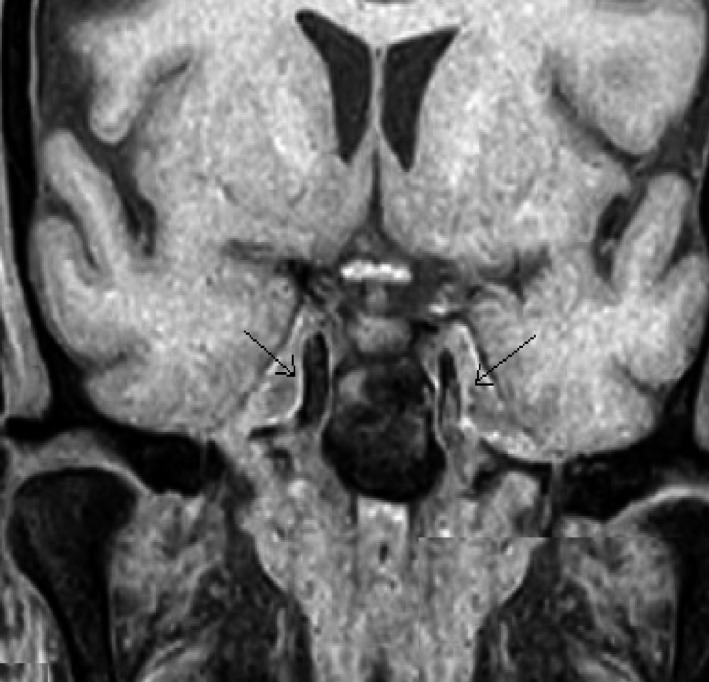
T1 SPIR sequence in MRI with contrast enhancement, showing the presence of internal carotid thrombosis, indicated by the arrow

After 2 days, a new MRI is performed, which highlight the presence of parietal, occipital, and frontal focal lesions and thrombosis of the left internal carotid artery (Figure 6). Given the poor response of the therapy, Amphotericin B is administered intrathecally 1.5 mg three times a week. In the following days, the clinical conditions worsened, with a negative response to therapy and poor glycemic compensation. The patient died 2 weeks after admission.

## 3 DISCUSSION

The starter symptoms of ROCM are compatible with those of periorbital cellulitis and sinusitis. They are characterized by facial and eye pain or blurry vision with facial numbness. The typical clinical symptoms of mucormycosis in predisposed subjects combine orbital inflammation, multiple cranial nerve palsies, eyelid edema, blepharoptosis, proptosis, unilateral periorbital facial pain, headache, acute ocular motility alteration, external or internal ophthalmoplegia, and acute vision loss. The pathognomonic sign of mucormycosis is a black necrotic eschar.

Fever is fluctuating and may be absent in more than half of the reported cases. The number of white blood cells is often high if the patient is immunocompetent. The radiological study before the operation is essential to define the extent of the disease. The CT study highlights the edematous mucosa and the possible destruction of the periorbital tissues, the inflammatory processes of the paranasal sinuses, and the bone margins. The CT study remains the best radiological method to study the phlogistic invasion of the disease. The rupture of bone margins occurs in the late stages of the disease after soft tissue necrosis. MRI is essential to evaluate the intracranial extension and involvement of the meninges of the pathology, the thrombosis of the internal carotid artery of cavernous portions, and the cavernous sinus thrombosis. Contrast‐enhanced MRI imaging may also show perineural invasion of infection. MRI imaging is more specific for the study of periorbital soft tissue infection than CT. In the early stages of the disease, patients may have a normal radiological examination with either CT or MRI. In the case of high suspicion of ROCM, endoscopic paranasal sinus surgery and biopsy sampling must always be performed.^17^ Radiological studies are important for assessing the extent of the disease, but the final diagnosis of mucormycosis occurs through the histopathological study that reveals the fungal invasion of the tissues.

**Figure 7:**
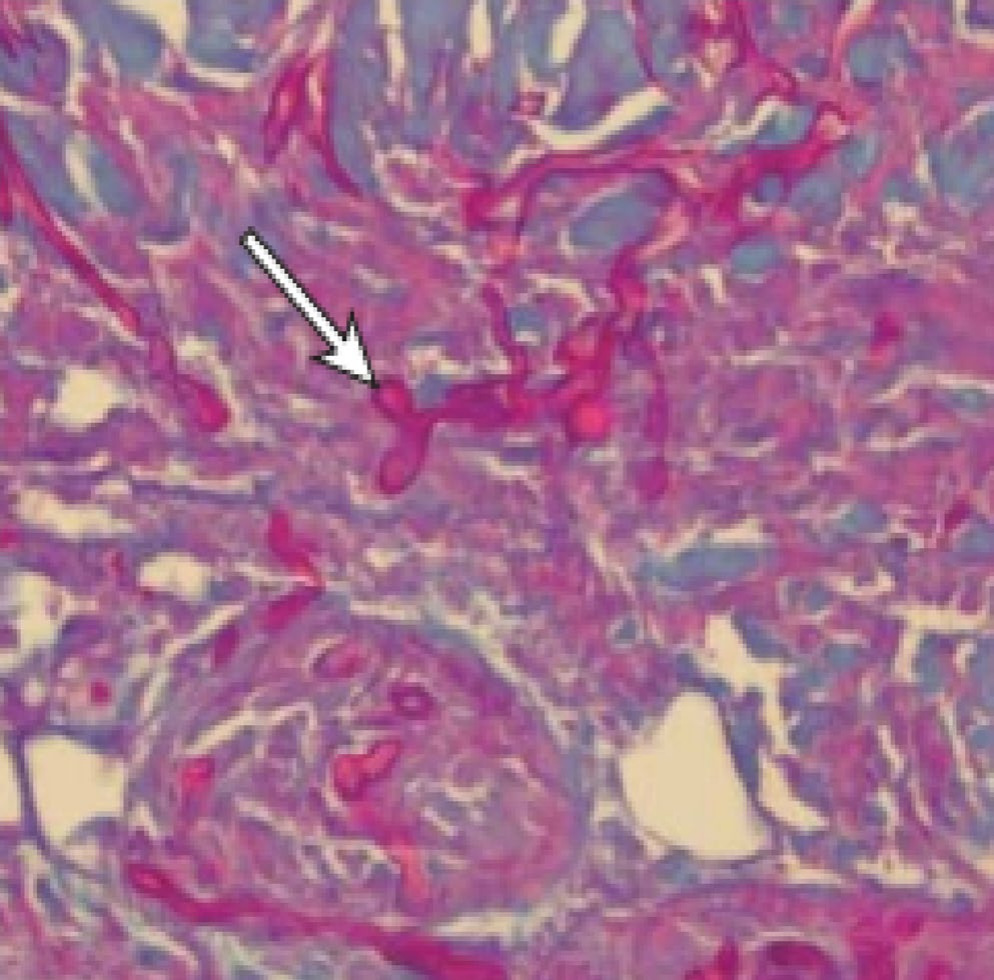
Periodic acid‐Schiff stain showed the elongated, pink fungal hyphae, irregularly branched and with rare septations (arrow)

The histological examination of Mucorales fungus in biopsy and their interpretation may be very difficult. These organisms are usually difficult to recognize on hematoxylin‐eosin stains. Gomori methenamine silver stains and Periodic acid‐Schiff are the best methods for a completely characterized appearance of the fungus. Despite this, it is possible to highlight only fragments, even with the use of cell wall coloring.^5^ The hyphae of the Mucorales are typical and consent for a presumptive diagnosis from clinical specimens. The hyphae are broad (5‐15 micron), irregularly branched, and have rare septations (Figure 7). This is in contrast with the hyphae of ascomycetous molds, such as Aspergillus, which are narrower (2‐5 micron), exhibit regular branching, and have many septations. The lack of regular settings can contribute to the fragile nature of hyphae and to the difficulty of growing mucormycosis agents from clinical samples, which is translated into a correct diagnosis of an untimely laboratory, like in our case at the first extemporaneous biopsy. Excessive movement of clinical samples can cause irreversible damage to hyphae.^18^

The main purpose of the therapy is to correct any predisposing risk factors, an aggressive surgery to eliminate the disease and a rapid antifungal treatment. Surgical debulking is carried out mainly by the ENT specialist, sometimes assisted by the neurosurgeon or ophthalmologist if there is intracranial or orbital delivery. Surgical debridement must be as aggressive as possible. Treatment with antifungal with intravenous amphotericin B should be started as quickly as possible. Liposomal formulations of amphotericin B are preferred to classic preparations of the antifungal agent because they are more tolerable in these already systemically compromised patients and have less side effect profiles. Liposomal amphotericin B also has fewer adverse reactions than the amphotericin B lipid complex, although the latter is much less expensive and, therefore, more readily available. No significant difference has been shown in efficacy between the three formulations of the drug, except from interruption of therapy from unbearable side effects. While amphotericin is typically administered intravenously, less is known about the use of intrathecal amphotericin B. We found in literature eight published cases of intrathecal amphotericin for treatment of mucormycosis, we found that seven of the eight previously published patients reported good long‐term outcome, suggesting that early intrathecal therapy warrants further consideration for difficult‐to‐treat intracranial fungal infections. In our case, we started intrathecal therapy when the patient's conditions were extremely compromised. We did not benefit from the therapy. In any case, more studies are needed about intrathecal therapy.^19^, ^20^, ^21^, ^22^, ^23^, ^24^, ^25^, ^26^ Other studies showed that posaconazole and isavuconazole as a second line agent could be used as salvage therapy in no response cases.^27^, ^28^

In a recent review about 929 cases of zygomycosis, survival was 3% (8 of 241 patients) for cases that were not treated, 61% (324 of 532) for cases treated with amphotericin B deoxycholate, 57% (51 of 90) for cases treated with surgery alone, and 70% (328 of 470) for cases treated with antifungal therapy and surgery.^29^, ^30^, ^31^

Rhinocerebral mucormycosis is rapidly letal, with a mortality rate of 50%‐85% even with an aggressive surgical debridement, an intravenous antifungal therapy, and a correction of predisposing risk factors.^32^, ^33^, ^34^

## 4 CONCLUSION

As a result of this, the ROCM is a rapidly progressive disease with an increase in the mortality rate if the fungus enters the cranial cavity. In all diabetic patients with sinus symptoms, headache, alterations of vision it is necessary to suspect a mucormycosis and carry out a careful radiological evaluation and a nasal endoscopy. Despite an early diagnosis, treatment for ROCM does not guarantee a cure from the disease that often ends with the patient's death.

## CONFLICT OF INTEREST

None declared.

## AUTHOR CONTRIBUTIONS

BG: was treating the patient. FGazia: is corresponding author, was preparing the manuscript. CG: Bibliography research and English translation. FP: performed follow‐up examinations. FC: developed the concept and design of the study. FF: The otorhin who first saw the patient and suspected mucormycosis. FGalletti: critically revised the manuscript and gave the approval of the final version.
